# Multiscale analysis of enantioselectivity in enzyme-catalysed ‘lethal synthesis’ using projector-based embedding

**DOI:** 10.1098/rsos.171390

**Published:** 2018-02-14

**Authors:** Xinglong Zhang, Simon J. Bennie, Marc W. van der Kamp, David R. Glowacki, Frederick R. Manby, Adrian J. Mulholland

**Affiliations:** 1 Physical and Theoretical Chemistry Laboratory, University of Oxford, South Parks Road, Oxford OX1 3QZ, UK; 2 Centre for Computational Chemistry, School of Chemistry, University of Bristol, Bristol BS8 1TS, UK; 3 School of Biochemistry, University of Bristol, Bristol BS8 1TD, UK; 4 Department of Computer Science, Merchant Venturers Building, Woodland Road, Bristol BS8 1UB, UK

**Keywords:** multiscale, coupled cluster, embedding

## Abstract

The action of fluoroacetate as a broad-spectrum mammalian pesticide depends on the ‘lethal synthesis’ of fluorocitrate by citrate synthase, through a subtle enantioselective enolization of fluoroacetyl-coenzyme A. In this work, we demonstrate how a projection-based embedding method can be applied to calculate coupled cluster (CCSD(T)) reaction profiles from quantum mechanics/molecular mechanics optimized pathways for this enzyme reaction. Comparison of pro-*R* and pro-*S* proton abstraction in citrate synthase at the CCSD(T)-in-DFT//MM level yields the correct enantioselectivity. We thus demonstrate the potential of projection-based embedding for determining stereoselectivity in enzymatic systems. We further show that the method is simple to apply, eliminates variability due to the choice of density functional theory functional and allows the efficient calculation of CCSD(T) quality enzyme reaction barriers.

## 1. Introduction

The citric acid cycle, also known as the tricarboxylic acid or Krebs cycle, consists of a series of chemical reactions involved in the production of molecular energy in all aerobic organisms [1]. The enzyme citrate synthase (enzyme classification: E.C. 2.3.3.1) catalyses the condensation of acetyl-coenzyme A (acetyl-CoA) and oxaloacetate to form citrate [2]. The action of citrate synthase can be divided into three separate steps. The initial step is the deprotonation of the acetyl moiety of acetyl-CoA, forming an enolate intermediate. This is followed by nucleophilic attack of the enolate intermediate on oxaloacetate, forming citryl-CoA. This stable intermediate is then hydrolysed by the enzyme to produce citrate and CoA.

**Figure 1.**
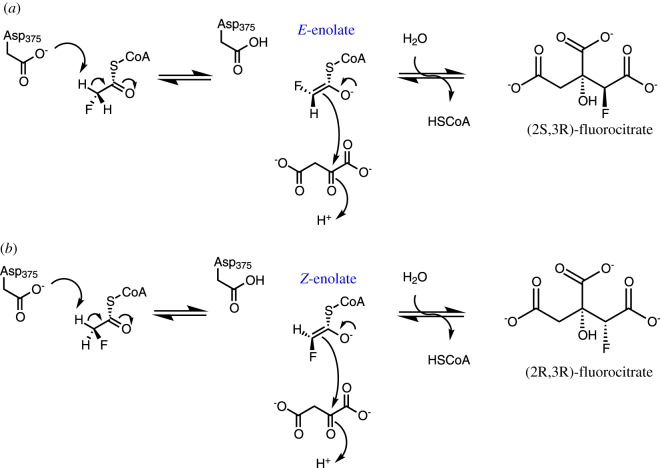
Conversion of fluoroacetyl-CoA to fluorocitrate by citrate synthase. Two stereoisomeric products are formed via either an (*a*) *E*- or (*b*) *Z*-enolate intermediate, obtained by deprotonation of acetyl-CoA by Asp375.

Fluoroacetate is a fluoro-substituted acetate that disrupts turnover of the citric acid cycle. Citrate synthase converts fluoroacetyl-CoA to fluorocitrate [3] using the same mechanism as in the formation of citrate from acetyl-CoA. Either the pro-*R* or the pro-*S* proton of the fluoroacetyl moiety can be abstracted in the first step, resulting in either the *E*- or *Z*-enolate, finally leading to two stereoisomers of fluorocitrate (figure 1). The more abundantly formed stereoisomer, (2*R*,3*R*)-fluoroacetate [4,5], is lethally toxic: it acts as a potent inhibitor of the enzyme aconitase [3], which catalyses the conversion of citrate to aconitate in the next step of the citric acid cycle. This results in halting the citric acid cycle and is thus the origin of ‘lethal synthesis’ by fluoroacetate [6,7]. Attempts to understand [7–9] and apply [10] the concept of ‘lethal synthesis’ in drug design date back to 1953 and understanding stereoselectivity is key to the mechanistic principles of fluoroacetate lethality.

Van der Kamp *et al.* [11] previously studied the enzymatic proton abstraction of fluoroacetate using high-level *ab initio* quantum mechanics/molecular mechanics (QM/MM) methods and concluded that *E*-enolate formation is favoured. In this study, geometries were optimized at the B3LYP/6-31+G(d)//CHARMM27 level and single-point energies were then calculated at the SCS-MP2 [12]/aug-cc-pVDZ//CHARMM27 level. This level was previously shown to agree with local coupled-cluster (LCCSD(T0)) QM/MM calculations for the same reaction with acetyl-CoA [13]. The calculated relative differences in the energies of the enolate intermediates (which are followed by the likely rate-limiting condensation step [14]) accurately reflected the observed stereoselectivity of the fluorocitrate products [15]. Here, we investigate this reaction using high-level electronic structure theory methods by using a projection-based embedding scheme. The advantages of using this new method compared to traditional electronic structure methods are that it is conceptually simple, flexible in its choice of high-level method and computationally efficient.

In our projection-based embedding scheme [16,17], the QM region is subdivided into regions that are treated at different QM levels. Atoms that are directly involved in the reaction are calculated with a high-level *ab initio* wavefunction-based technique, such as coupled cluster with singles, doubles and perturbative triples (CCSD(T)). The more distant surrounding chemical moieties that are not directly involved in the reaction, but could nonetheless affect the electronic structure of the reacting centres, are treated with more approximate (and cheaper) density functional theory (DFT) methods. The role of projection-based embedding is to couple the electronic structure methods; this results in a CCSD(T) calculation polarized by a DFT environment. By using correlated *ab initio* wavefunctions in the embedding scheme, we eliminate the functional sensitivity of the DFT method used for the region not chemically critical to the reaction. The projection-based embedding technique has the added advantage that sources of error can be easily interpreted, and systematically improved [17]. The system treated with CCSD(T)-in-DFT can then be further incorporated into the traditional QM/MM treatment of the wider protein environment to give a complete picture of the enzyme. Such multi-scale embedding schemes benefit greatly from increased computational efficiency compared with full-scale high-level *ab initio* quantum calculation while maintaining reasonable accuracy. To further decrease computational cost, we use a basis-set truncation technique that removes basis functions that are distant from the active region [18,19], automatically determined by a single parameter. Although the projection-based embedding scheme has been applied to various chemical systems [20,21], it has only recently been applied in QM/MM modelling of an enzyme reaction [22]. In this work, we extend the application of this technique to the study of enzyme enantioselectivity and show how the method can provide chemical insights.

## 2. Computational methods

To a reasonable approximation, the deprotonation of fluoroacetyl-CoA can be defined by the reaction coordinate:

2.1 $$rc=d({\text{C}}_{\mathrm{FaCoA}}\text{H})-d({\text{O}}_{\mathrm{Asp}375}\text{H}),$$

which is the difference between the C−H bond length of the fluoromethyl moiety of fluoroacetyl-CoA and the O−H bond length between the oxygen atom on Asp375 and the abstracted hydrogen. This reaction coordinate was shown to accurately represent the reaction pathway [13].

Previously, the geometries used in this study are taken from van der Kamp *et al.* [11], where a model of the reactant complex was made by replacing acetyl-CoA with fluoroacetyl-CoA and *R*-malate by oxaloacetate in the crystal structure of chicken citrate synthase (PDB code: 4CSC) [23]. In this work, transition state conformations were generated from this reactant state model using QM/MM umbrella sampling at the AM1//CHARMM27 level, and subsequently, iterative QM/MM optimizations at the B3LYP/6-31+G(d)//CHARMM27 level were performed in both directions to obtain structures between *rc*=−1.4 Å and *rc*=1.4Å in 0.1 Å increments [11]. In these geometry optimizations, the side-chain of Asp375, the methylthioester portion of fluoroacetyl-CoA and oxaloacetate were chosen to be the QM region. The inclusion of oxaloacetate in the QM region, despite it being a spectator molecule in the deprotonation, is known to decrease the barrier height by approximately 3 *kcal* *mol*^−1^ [13], likely due to the inclusion of oxaloacetate polarization by the enzyme active site and its influence on the enolization reaction [24]. The energies for these reaction profiles were calculated at the spin-component-scaled (SCS)-MP2 QM level using the aug-cc-pVDZ basis set, in conjunction with the CHARMM27 MM potential. These traditional or ‘canonical’ SCS-MP2//MM energies were shown to agree well with the experimental results [11,15].

**Figure 2.**
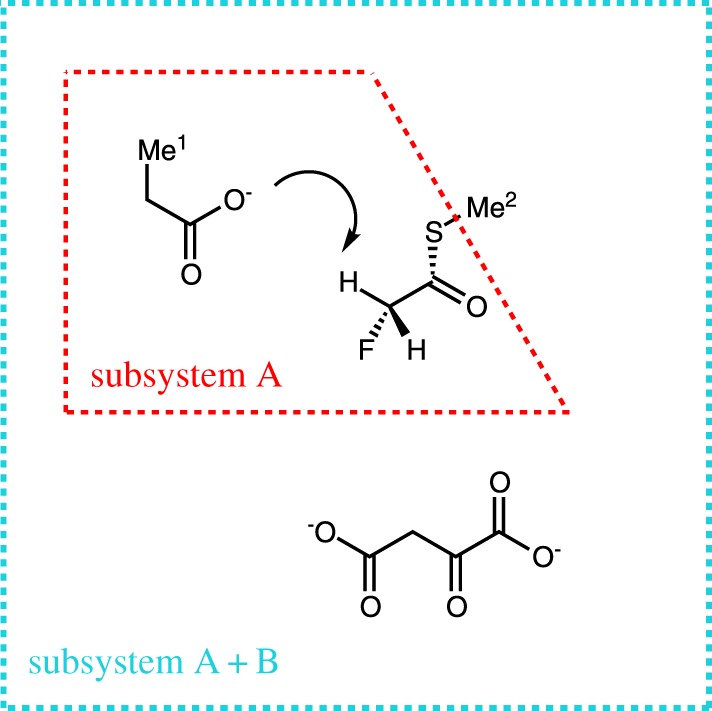
Representation of the QM region of the QM/MM partitioning in the deprotonation of fluoroacetyl-CoA by citrate synthase. One hydrogen atom on *Me*^1^ side chain is treated as a link atom linking the Asp375 residue to the MM region of the rest of the enzyme. Similarly, a hydrogen atom on *Me*^2^ was used to link the fluoroacetyl-S-Me moiety to the rest of coenzyme A in the MM region.

All calculations in this work used the aug-cc-pVDZ basis set [25–27] for all atoms in subsystems A and B. QM/MM calculations were performed using the LDA [28,29], PBE [30], PBE0 [31], B3LYP [32] and BH&HLYP (or BHLYP) [33] functionals, as well as Hartree–Fock (HF) theory, for the QM subsystem. For embedding calculations, single-point WF-in-DFT//MM calculations were performed using the Molpro 2015.1 software package [34,35]. The QM region consisted of an acetate group representing the Asp375 residue of the enzyme citrate synthase, methylfluorothioacetate representing fluoroacetyl-CoA, and a molecule of oxyloacetate (figure 2). We further divide this QM region into two subsystems. Atoms directly involved in the deprotonation reaction (those defined in the reaction coordinate above) and all atoms up to two bonds away from them are included in subsystem A to ensure that enough electrons are included for the WF correlation treatment. The oxaloacetate molecule is included in subsystem B because its QM treatment has been shown to influence the reaction profile significantly [13]. Subsystem A was treated using MP2 [36], SCS-MP2 [12] and CCSD(T) [37,38] methods; it contains 80 electrons for correlation treatment, and the sulfur valency in the embedding calculations was satisfied by including the covalent bond with the methyl group. Subsystem B was treated with the previously discussed functionals. The 1s, 2s and 2p electrons on the sulfur atom were treated as core electrons and were not correlated in the post-HF methods; all other electrons were correlated. Acceleration of the wavefunction methods was achieved with basis truncation [18] using the default parameter with a value of 10^−4^. These embedded single-point WF-in-DFT energies were performed in the field of MM point charges and MM internal energies as well as QM/MM van der Waals interactions were added from the previous B3LYP/6-31+G(d)//CHARMM27 calculations [11] to yield the final energies for the enzymatic reaction.

## 3. Results and discussion

**Figure 3.**
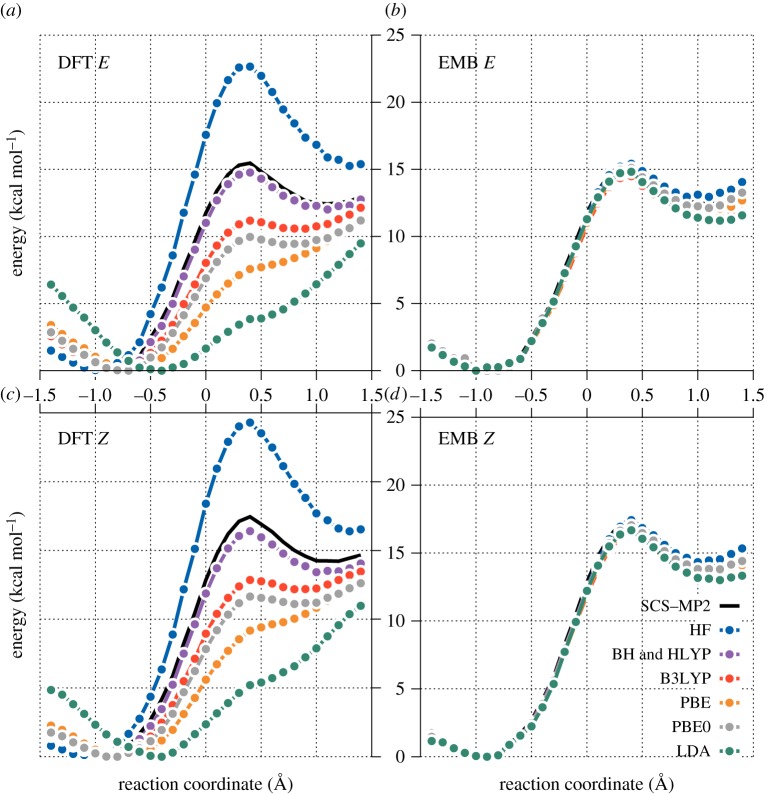
Canonical HF/MM, DFT//MM and SCS-MP2//MM and SCS-MP2//MM with DFT (or HF) embedding energy profiles for enolate formation. (*a*) Canonical QM/MM *E*-enolate formation. (*c*) Canonical QM/MM *Z*-enolate formation. (*b*) Embedded SCS-MP2//MM *E*-enolate formation. (*d*) Embedded SCS-MP2//MM *Z*-enolate formation. The aug-cc-pVDZ basis set was used throughout.

**Figure 4.**
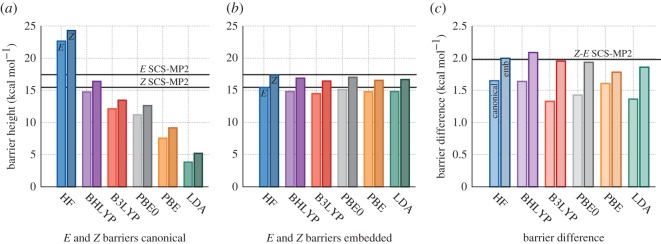
(*a*) Canonical DFT//MM (or HF//MM) barrier heights for *E* and *Z*-enolates. (*b*) SCS-MP2//MM barriers with DFT (or HF) embedding. (*c*) Barrier height differences between enolates for canonical and embedded results. The black lines indicate the canonical SCS-MP2//MM benchmark values for the reference profiles used.

Experimentally, it was determined that fluoroacetyl-CoA is converted by citrate synthase into (2*R*,3*R*)-fluorocitrate as the major product, with its stereoisomer (2*S*,3*R*)-fluorocitrate amounting to 2–3% of the product [15]. Using transition state theory, this corresponds to a difference in activation energy of around 2.06–2.30 *kcal* *mol*^−1^. Previous computational studies at the SCS-MP2//MM level indicated that the difference in reaction energies for *E*- and *Z*-enolate formation predict this difference accurately [11]. To assess the performance of different density functionals on the reaction profiles of the *E*- and *Z*-enolate formations, we performed single-point QM/MM calculations using various DFT functionals on the full QM region (subsystems A + B) with full basis set. These functionals included a primitive local density approximation functional (LDA), two general gradient approximation functionals (BP86 and PBE) and three hybrid functionals (PBE0, B3LYP and BHLYP). We also included the HF method for comparison. Although the energies for the *Z*-enolate (leading to the minor product) were calculated to be higher than the corresponding *E*-enolate within each method, the relative energies and shapes of the energy profiles produced by different DFT methods vary widely (figure 3*a*,*c*). For both enolates, the three hybrid functionals (BHLYP, B3LYP and PBE0) predict a minimum (albeit shallow for B3LYP and PBE0). The pure functionals LDA and PBE failed to predict stable enolate formation (PBE and BP86 were found to be nearly identical). These different DFT methods gave a large spread in the energy of the transition states (*ca* 11 *kcal* *mol*^−1^) in both enolate formations as is shown in figure 4. HF is known to overestimate the barrier heights of reactions [39,40] including proton abstraction from acetyl-CoA by citrate synthase [13]; with HF included the barrier heights span a range of *ca* 19 *kcal* *mol*^−1^. These results demonstrate that the barrier height is strongly dependent on the choice of the functional, which makes it difficult to assess the accuracy of QM/MM results without *a priori* knowledge.

Applying projection-based embedding with SCS-MP2 in subsystem A removes much of the variation between DFT functionals in the energy profiles of both enolate formations (figure 3*b*,*d*). For both the *E*- and *Z*-enolates, all the SCS-MP2-embedded DFT and HF methods now agree quantitatively with the canonical SCS-MP2 reference results. The agreement is particularly good (within 1 *kcal* *mol*^−1^) for reaction coordinate values from −1.5Å to 0.7 Å which include the reactant state minimum and the transition state maximum (also highlighted in figure 4). These results show that it is possible to largely eliminate the variability of the QM method in QM/MM calculations by using a high-level electronic structure method on a subset of atoms chosen using basic chemical knowledge. The enolate intermediate minimum shows, however, a larger variation between the different embedding methods, displaying a maximum difference of approximately 2 *kcal* *mol*^−1^ for the *E*-enolate and approximately 1.5 *kcal* *mol*^−1^ for the *Z*-enolate (with HF included). This maximum difference is between HF and the LDA functional (possibly because these two methods do not capture oxaloacetate densities well at the mean field level; figure 3), which are two methods that would not normally be used in QM/MM studies because of their known deficiencies. The WF-embedded generalized gradient approximation and hybrid functionals (PBE, BHLYP, B3LYP and PBE0) agree reasonably well for the reaction energies (within 0.6 *kcal* *mol*^−1^ for both enolates). Importantly, all the embedded calculations result in the same locations for the reactant and enolate intermediate minima and the transition state maximum. This indicates a significant improvement compared to the QM/MM calculations with DFT methods for the full QM region (figure 3), which show not only a significant variation in the energies but also predict different locations of the minima and maxima on the profiles.

The energy differences in the QM/MM activation barriers of *E*- and *Z*-enolate formation vary between functional (figure 4*a*), and crucially are different from the fully canonical SCS-MP2 results (figure 4). In this case, the BHLYP functional performs best, with satisfactory results for both the absolute barriers and barrier difference between *Z*- and *E*-enolate formation. This is likely a fortuitous combination of the large fraction of exact exchange in this functional and the particular enzyme–reactant conformation used here (see further details in [13]). The barrier difference of the LDA functional between −1Å and 0.4Å coordinates deviates from the benchmark by 0.63 *kcal* *mol*^−1^; this error is reduced to 0.12 *kcal* *mol*^−1^ with our embedding scheme (figure 4*b*). The other functionals also demonstrate improvement when used for embedding, with HF, B3LYP and PBE0 showing 0.03 *kcal* *mol*^−1^ differences to the canonical SCS-MP2 benchmark. From figure 4*c*, it can be seen that for embedding the inclusion of exact exchange appears to result in the best agreement with the full SCS-MP2 calculations; this is most likely due the subsystem B densities being improved by its inclusion. We note that the energy differences in this case are very subtle (with differences between canonical and embedding results ranging between 0.3 and 1.2 *kcal* *mol*^−1^), but embedding consistently improves the estimate for the difference in barrier, which is crucial to estimate the degree of enzyme enantioselectivity. The overall improvement of the barrier difference with embedding shows that our projection embedding coupled with QM/MM does not just reliably calculate single-point energies, but can also be used to accurately distinguish subtle (bio)chemical selectivity.

**Figure 5.**
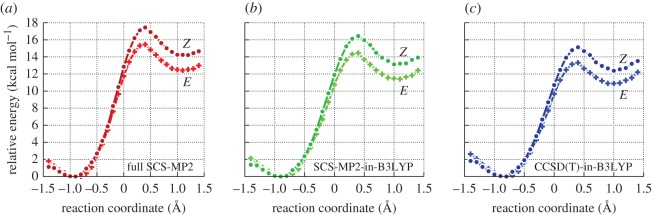
QM/MM reaction profiles for various methods calculated with the aug-cc-pVDZ basis set. (*a*) All-electron (canonical) SCS-MP2 reaction profiles with full basis set. (*b*) SCS-MP2-in-B3LYP//MM with basis set truncation. (*c*) CCSDT(T)-in-B3LYP//MM with basis set truncation.

As projection-based embedding only modifies the core Hamiltonian, we are able to straightforwardly investigate how applying different WF-based methods to subsystem A affects the energy profiles for *E*- and *Z*-enolate formation. We compare WF-in-B3LYP embedding calculations where the WF method is MP2, SCS-MP2 or CCSD(T). MP2 activation and reaction energies are, respectively, approximately 3.1 *kcal* *mol*^−1^ and approximately 1.9 *kcal* *mol*^−1^ lower than the referenced energy profiles for both enolates (results not shown), as expected for this method from previous computational studies of the same reaction [13] and of other enzymatic systems [41,42]. SCS-MP2 embedding results agree well with the canonical SCS-MP2 profiles (figure 5); the activation and reaction energies for both enolate formations are all within 1 *kcal* *mol*^−1^ of the canonical results, indicating that SCS-MP2-in-B3LYP (or SCS-MP2-in-DFT in general, as shown in figure 3) embedding can yield chemically accurate results while reducing computational cost. CCSD(T)-in-B3LYP yields activation and reaction energies that are somewhat lower than for SCS-MP2-in-B3LYP, and the differences between the *E*- and *Z*-enolate barrier and reaction energies at this level are lower as well (1.8 and 1.5 *kcal* *mol*^−1^, respectively; still consistent with experiment [15]). Lower activation and reaction energies are expected based on our previous results [22], which showed that CCSD(T) embedded results had lower barriers (0.6 *kcal* *mol*^−1^) and reaction energies (by 0.6 *kcal* *mol*^−1^) than SCS-MP2 for the reaction with acetyl-CoA. The decrease in the barrier and reaction energies by increasing the level of WF theory to CCSD(T) cannot directly be related to better agreement with experiment (as the subsequent step, condensation, is likely to be rate-limiting for citryl-CoA formation [14]). It does, however, show that there is scope with projection-based embedding to choose more advanced correlation methods in enzyme reaction studies, to provide confirmation that perturbation methods such as (SCS-)MP2 are returning accurate results.

## 4. Conclusion

We have extended the method of projection-based WF-in-DFT embedding in a wider MM enzyme environment to the modelling of enzyme stereoselectivity. The advantages of this multiscale approach are threefold: it eliminates the variability of calculated barriers of traditional DFT; the simple modification of a core Hamiltonian for the active system allows the usage of various *ab initio* methods; and it only requires minimal chemical knowledge to perform the calculations. Our methods produce energy profiles that give quantitative agreement with experimentally observed product ratios and with more computationally demanding calculations. This work shows that the use of our embedding method, in conjunction with QM/MM reaction modelling, is an efficient and accurate approach for predicting the outcome of stereoselective enzyme reactions, thus opening up many possibilities for studying subtle selective reactions catalysed by enzymes.

## Supplementary Material

SI-multiscale-analysis-enantioselectivity.pdf

## Data accessibility

The electronic supplementary material includes MOLPRO input files required to generate all of the data reported in this paper.
