# Supplementary material for: Multiscale analysis of enantioselectivity in enzyme-catalysed ‘lethal synthesis’ using projector-based embedding
Source: R Soc Open Sci. 2018 Feb 14;5(2):171390. doi: 10.1098/rsos.171390 (PMC5830745; doi:10.1098/rsos.171390)
Supplement: SI-multiscale-analysis-enantioselectivity.pdf [file rsos171390supp1.pdf]

Multiscale analysis of enantioselectivity in enzyme-catalysed 'lethal synthesis' using projector-based embedding

Xinglong Zhang,[1] Simon J. Bennie,[2] Marc W. van der Kamp,[2,3] David R. Glowacki,[2,4] Frederick R. Manby[2] and Adrian J. Mulholland[2]

[1] *Physical and Theoretical Chemistry Laboratory, University of Oxford, South Parks Road, Oxford, OX1 3QZ, UK*
[2] *Centre for Computational Chemistry, School of Chemistry, University of Bristol, Bristol BS8 1TS, UK*
[3] *School of Biochemistry, University of Bristol, Bristol BS8 1TD, UK*
[4] *Department of Computer Science,Merchant Venturers Building,Woodland Road,Bristol BS8 1UB UK*

1 Example input file for the generation of Projection-based embedding profiles with QM/MM

```
memory,500, m

geomtyp=xyz
nosym
noorient

geometry={
C1      −11.443994    13.530871     6.095726
C2      −12.615096    13.486453     5.089573
O3      −12.671857    12.450378     4.348016
O4      −13.430702    14.447066     5.075337
H5      −10.650551    12.835197     5.811858
H6      −11.818585    13.266354     7.090616
S7      −10.925788    11.825331     1.175627
C8      −12.619528    11.279574     1.352945
O9      −12.934846    10.086439     1.209463
C10     −13.531530    12.315282     1.834446
F11     −14.862996    11.935817     1.636166
H12     −13.361275    13.300507     1.382042
H13     −13.258648    12.428756     3.079395
C14     −10.092723    10.245621     1.547309
H15      −9.020619    10.432246     1.456436
H16     −10.359972     9.488339     0.810288
C17     −14.072663    13.444345    −2.071347
O18     −14.232385    14.586944    −1.555392
O19     −14.958103    12.697051    −2.546950
C20     −12.612932    12.910579    −2.146510
O21     −11.667764    13.680893    −2.045564
C22     −12.391333    11.432316    −2.358710
C23     −11.955042    11.047501    −3.788249
O24     −11.685646     9.818408    −3.973958
O25     −11.886566    11.944357    −4.665387
H26     −11.586959    11.120745    −1.683309
```

```
H27      -13.291369    10.861090    -2.119560
H28      -11.035825    14.561474     6.157823
H29      -10.336244     9.927175     2.565521
}
#Import the MM point charges for QM/MM, in this case a file called lattice
lattice,infile=lattice.

#Define the basis set.
basis={
default,aug-cc-pvdz
}

#Run initial low level method, any functional can be used here.
{ks,b3lyp,direct;wf,156,1,0}
#localise the orbitals.
{ibba,bonds=1,iborth='ZBD'}
#Run the projection embedding code.
{embed,proj,n_orbitals=40,aotrunc,denkeep=0.0001;atoms,C8,O9,C10,H12,H13,F11,O3,C2,O4
#Run the post embedding low-level method to determine type-in-type error.
{ks,b3lyp,direct;wf,80,1,0}
#Run pre-correlation mean field , usually HF.
{hf,direct;wf,80,1,0}
#Run the High-level correlation method.
{ccsd(t);core,5;wf,80,1,0}
```
